# Genetic Diversity, Inbreeding Level, and Genetic Load in Endangered Snub-Nosed Monkeys (*Rhinopithecus*)

**DOI:** 10.3389/fgene.2020.615926

**Published:** 2020-12-15

**Authors:** Weimin Kuang, Jingyang Hu, Hong Wu, Xiaotian Fen, Qingyan Dai, Qiaomei Fu, Wen Xiao, Laurent Frantz, Christian Roos, Tilo Nadler, David M. Irwin, Linchun Zhou, Xu Yang, Li Yu

**Affiliations:** ^1^State Key Laboratory for Conservation and Utilization of Bio-Resource in Yunnan, School of Life Sciences, Yunnan University, Kunming, China; ^2^Key Laboratory of Vertebrate Evolution and Human Origins of Chinese Academy of Sciences, IVPP, CAS, Beijing, China; ^3^Center for Excellence in Life and Paleoenvironment, Chinese Academy of Sciences, Beijing, China; ^4^Beijing College of Life Science, University of Chinese Academy of Sciences, Beijing, China; ^5^Institute of Eastern-Himalaya Biodiversity Research, Dali University, Dali, China; ^6^School of Biological and Chemical Sciences, Queen Mary University of London, London, United Kingdom; ^7^The Palaeogenomics and Bio-Archaeology Research Network, Department of Archaeology, University of Oxford, Oxford, United Kingdom; ^8^Gene Bank of Primates and Primate Genetics Laboratory, German Primate Center, Leibniz Institute for Primate Research, Göttingen, Germany; ^9^Wildlife Consultant, Ninh Binh, Vietnam; ^10^Department of Laboratory Medicine and Pathobiology, University of Toronto, Toronto, ON, Canada; ^11^Lushui Management and Conservation Branch of Gaoligong Mountain National Nature Reserve, Nujiang, China; ^12^Lushui Forestry and Grassland Council, Nujiang, China

**Keywords:** snub-nosed monkeys, population genomics, genetic diversity, inbreeding, genetic load, population decline

## Abstract

The snub-nosed monkey genus (*Rhinopithecus*) comprises five closely related species (*R. avunculus, R. bieti, R. brelichi, R. roxellana*, and *R. strykeri*). All are among the world's rarest and most endangered primates. However, the genomic impact associated with their population decline remains unknown. We analyzed population genomic data of all five snub-nosed monkey species to assess their genetic diversity, inbreeding level, and genetic load. For *R. roxellana, R. bieti*, and *R. strykeri*, population size is positively correlated with genetic diversity and negatively correlated with levels of inbreeding. Other species, however, which possess small population sizes, such as *R. brelichi* and *R. avunculus*, show high levels of genetic diversity and low levels of genomic inbreeding. Similarly, in the three populations of *R. roxellana*, the Shennongjia population, which possesses the lowest population size, displays a higher level of genetic diversity and lower level of genomic inbreeding. These findings suggest that although *R. brelichi* and *R. avunculus* and the Shennongjia population might be at risk, it possess significant genetic diversity and could thus help strengthen their long-term survival potential. Intriguingly, *R. roxellana* with large population size possess high genetic diversity and low level of genetic load, but they show the highest recent inbreeding level compared with the other snub-nosed monkeys. This suggests that, despite its large population size, *R. roxellana* has likely been experiencing recent inbreeding, which has not yet affected its mutational load and fitness. Analyses of homozygous-derived deleterious mutations identified in all snub-nosed monkey species indicate that these mutations are affecting immune, especially in smaller population sizes, indicating that the long-term consequences of inbreeding may be resulting in an overall reduction of immune capability in the snub-nosed monkeys, which could provide a dramatic effect on their long-term survival prospects. Altogether, our study provides valuable information concerning the genomic impact of population decline of the snub-nosed monkeys. We revealed multiple counterintuitive and unexpected patterns of genetic diversity in small and large population, which will be essential for conservation management of these endangered species.

## Introduction

**Figure 1:**
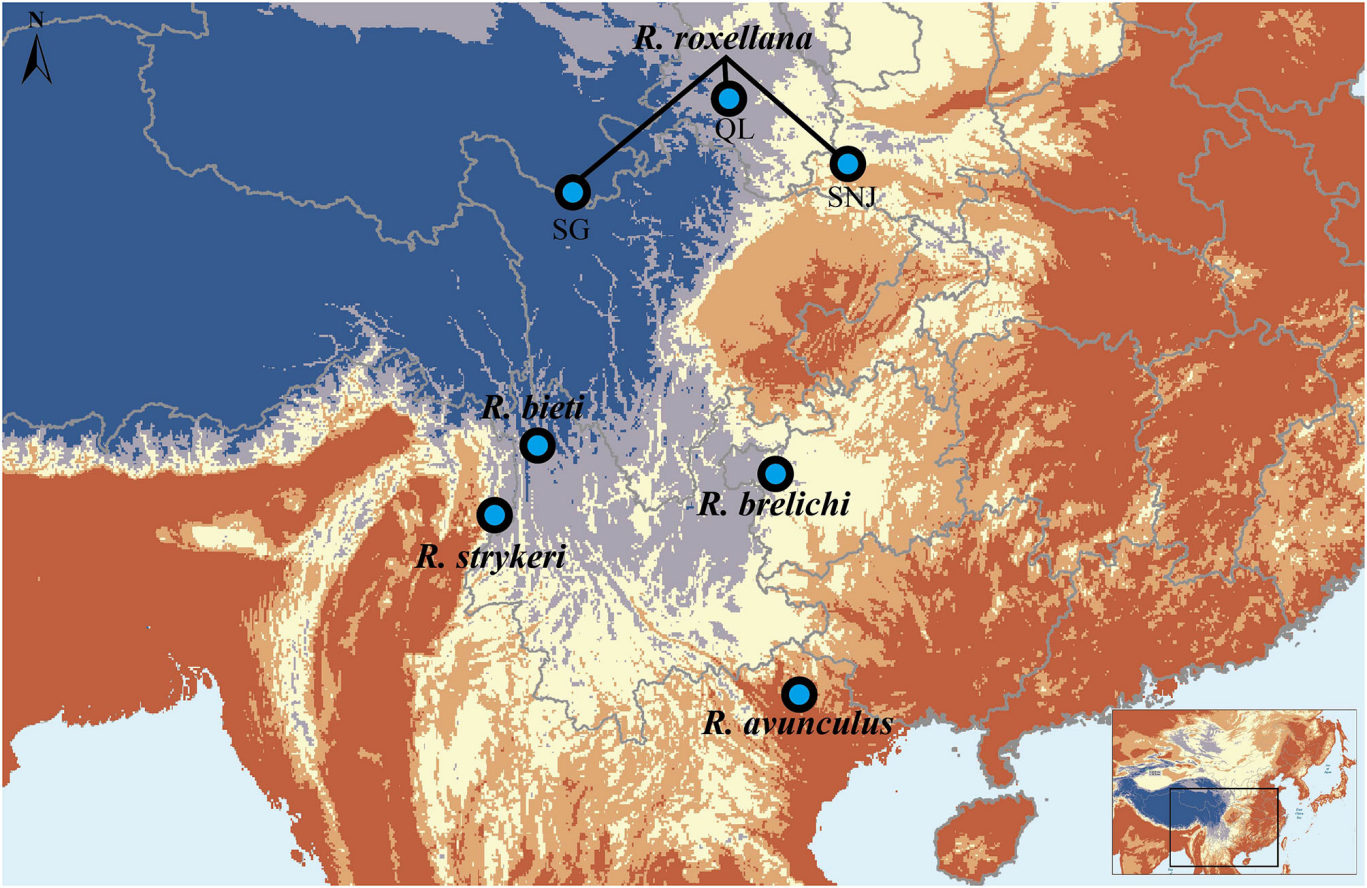
Geographic distribution of the *Rhinopithecus* species. The map is drawn using ESRI ArcGIS 10.2.

The snub-nosed monkey genus (*Rhinopithecus*) comprises five closely related species. The golden snub-nosed monkey (*R. roxellana*), Yunnan snub-nosed monkey (*R. bieti*), and Guizhou snub-nosed monkey (*R. brelichi*) are endemic to China, while the Tonkin snub-nosed monkey (*R. avunculus*) is distributed in northern Vietnam, and the Myanmar/Nujiang snub-nosed monkey (*R. strykeri*) inhabits northern Myanmar and the Nujiang prefecture in China (Geissmann et al., 2011; Liedigk et al., 2012; Ma et al., 2014; Meyer et al., 2017) (Figure 1). Currently, all five species are classified as endangered or critically endangered on the International Union for Conservation of Nature (IUCN) Red List.

Fossil records indicate that the snub-nosed monkeys were widely distributed across East Asia during the Late Pleistocene and Early Holocene (Han, 1982; Jablonski and Pan, 1988; Jablonski, 1998). Environmental changes during the Holocene, however, led to habitat loss and fragmentation for all five species, and this process was likely accelerated by increasing human activities over the last 400 years (Li et al., 2002; Nuchel et al., 2018). In fact, recent field surveys indicated that the population sizes in these species were extremely low. For example, there are only 22,500 individuals of *R. roxellana*, the most numerous species, which are isolated in three fragmented populations in the Minshan and Qionglai mountains (SG; Sichuan/Gansu provinces), the Qinling mountain (QL; Shanxi province), and the Shennongjia National Nature Reserve (SNJ; Hubei province) (Quan and Xie, 2002; Liu et al., 2015; Li et al., 2018). Other species, such as *R. bieti* and *R. strykeri* are only ~3,000 (Li et al., 2018; Zhao et al., 2019) and ~950 individuals (Meyer et al., 2017; Ren et al., 2017; Yang et al., 2019), respectively, and there are fewer than 400 and 200 individuals of most endangered species *R. brelichi* (Guo et al., 2020) and *R. avunculus* (Nadler, 2018) left in the wild, respectively.

These dramatically low population sizes, if maintained long enough, may cause loss of genetic diversity, increase of inbreeding, and accumulation of deleterious mutations (genetic load), all of which can reduce adaptive potential and dramatically increase risk of extinction (Hansson and Westerberg, 2002; Frankham, 2005; Heller and Zavaleta, 2009; Jump et al., 2009). These issues, however, can be mitigated with appropriate conservation management that takes into account genetic diversity (Frankham, 2005). Yet, despite the fact that many studies have investigated the taxonomy, phylogeography, and deep evolutionary history of the snub-nosed monkeys based on mitochondrial DNA and microsatellites (Zhang and Ryder, 1997; Li et al., 2004, 2007; Liu et al., 2007, 2009, 2015; Chang et al., 2012; Liedigk et al., 2012; Yang et al., 2012; Kolleck et al., 2013; Hong et al., 2017), and more recently based on whole genome sequencing (Zhou et al., 2014, 2016; Yu et al., 2016; Kuang et al., 2019), little is known about the genetic diversity and levels of inbreeding in these populations.

To explore the genetic diversity, inbreeding level, and genetic load in these species, we analyzed 62 genome sequences representing individuals from all five snub-nosed monkeys. Our study provides valuable information concerning the genomic impact of population decline of the snub-nosed monkeys. In particular, we unravel unexpected patterns of genetic diversity in small and large population, which will be essential for conservation management of these endangered species.

## Materials and Methods

### Data Collection and Genome Sequencing

Ninety-five published genomic data from all five snub-nosed monkeys, including 57 *R. roxellana*, 28 *R. bieti*, 1 *R. avunculus*, 5 *R. brelichi*, and 4 *R. strykeri* (Zhou et al., 2014, 2016; Yu et al., 2016; Kuang et al., 2019) were downloaded from the National Center for Biotechnology Information (NCBI). An additional, seven *R. bieti*, three *R. strykeri*, and one *R. avunculus* were sequenced in this study (Supplementary Table 1). A bone (R.strykeri-7) and two skin (R.strykeri-5 and R.strykeri-6) samples of *R. strykeri* individuals from the Gaoligong Mountain National Nature Reserve were provided by the Nujiang Forest Public Security Bureau and the Institute of Eastern-Himalaya Biodiversity Research, respectively. A skin (R.avunculus-2) sample of *R. avunculus* from the Ba Be National Park of northern Vietnam was provided by the Gene Bank of Primates at the German Primate Center. DNA samples of *R. bieti* from the Baima Mountain National Nature Reserve were provided by the Animal Branch of the Germplasm Bank of wild Species of Chinese Academy of Science. With the addition of these new samples, we obtained a total of 106 population genomes (average 12.02-fold coverage) representing all five snub-nosed monkeys. The genomic sequences from *Pygathrix nemaeus* (red shanked douc langur) and *Macaca mulatta* (rhesus macaque; SRS748669; Zhang et al., 2014) were used as outgroups. The DNA sample of *Pygathrix nemaeus* obtained from the Animal Branch of the Germplasm Bank of wild Species of Chinese Academy of Science was used to generate the genomic sequences (32.13-fold coverage).

Genomic DNAs from the skin and bone samples of *R. strykeri* and *R. avunculus* were extracted in the ancient DNA extraction facility of the Institute of Vertebrate Paleontology and Paleoanthropology (IVPP), Chinese Academy of Sciences, Beijing, China (for details, see Supplementary Notes). Sequencing libraries were produced using a double stranded library preparation protocol (Meyer and Kircher, 2010; Kircher et al., 2012) with uracil-DNA-glycosylase (UDG) and endonuclease (Endo VIII) treated (for details see Supplementary Notes). Sequencing libraries were sequenced using paired-end (PE) 150-bp reads on an Illumina HiSeq X-ten platform. Reads were demultiplexed by allowing one mismatch on each pair of reads. A modified version of SeqPrep (John, 2011) were used to collapse the PE reads (at least 11-bp overlap with one mismatch allowed), and base quality in the overlapping regions was set to the highest Phred score. Collapsed pairs were aligned using BWA samse v 0.6.1 (Li and Durbin, 2009) after stripping adapters. All duplicates were removed with bam-rmdup (https://github.com/mpieva/biohazard-tools) by keeping only the read for each set of duplicates with the highest quality bases.

Sequencing libraries based on high-quality genomic DNAs from *R. bieti* and *P. nemaeus* were constructed with an insert size of 350 bp and sequenced on an Illumina HiSeq 2500 platform using PE 100–150 bp reads. All newly sequenced genomic data generated for this study were deposited on the Short Read Archive (SRA) database under project number: PRJNA616055.

### Reads Alignment, Genotype Calling, and Filtering

A high-quality reference genome is needed for population genomic studies. The published chromosome-level genome of the golden snub-nosed monkey (Wang et al., 2019a) was used as the reference genome here. Reads were aligned to the reference genome with BWA-MEM (Li and Durbin, 2009) and SAMtools v.1.3 to generate BAM files (Li et al., 2009). Aligned reads were realigned around inserts/deletions (INDELs) using GATK v3.8 indelRealigner (Mckenna et al., 2010) and duplicate reads were marked using Picard v2.10.3 (https://broadinstitute.github.io/picard).

We called single nucleotide polymorphism (SNPs) with GATK v3.8 HaplotypeCaller (Mckenna et al., 2010). Raw SNPs were then filtered for quality and depth using the following criteria: Variants failing the recommended GATK hard filters (QD < 2.0 || FS > 60.0 || MQ < 40.0 || ReadPosRankSum < -8.0 || SB >= −1.0 || DP < 3), low Phred score (QUAL of <30), missingness above 20%, allele frequency <5% and sites identified within CpG islands using CpGIScan software (Jian et al., 2017) with default parameter values were excluded. Only biallelic autosomal SNPs were analyzed in this study (i.e., triallelic SNPs and SNPs with mapping to the chromosomes X and Y were excluded).

### Close Kinship Analyses

Given that the close relationship of samples has the potential to bias the heterozygosity, inbreeding, and genetic load results, so the kinship analyses among the collected genome sequences from the individuals for each species were performed with the Kinship-based Inference for Genome-wide association studies (KING) (Manichaikul et al., 2010) to remove the potential consanguineous individuals. Kinship coefficient was estimated with the “-kinship” command from KING v.2.2.5, which reflects the proportion of SNPs with identical state (IBS0, identity by state zero) between individuals. Negative coefficients indicate unrelated relationships, while positive coefficients indicate genealogy links between individuals. Nonconsanguineous individuals (kinship coefficients <0) were used in the subsequent analyses (Supplementary Table 1).

### Whole Genome Heterozygosity and Nucleotide Mismatches Analyses

Whole genome heterozygosity (*He*) and pairwise nucleotide divergence were computed in 100-kb sliding-window size with no step using variant cell format (VCF) tools (Danecek et al., 2011) and in-house Perl scripts, respectively.

### Runs of Homozygosity and Inbreeding Analyses

Run of homozygosity (ROHs) are contiguous homozygous segments of the genome where the two haplotypes inherited from the parents are identical (Gibson et al., 2006), which can be used to estimate inbreeding level (Keller et al., 2011). Since recombination events interrupt lengthy genome segment, thus long ROHs (long-ROHs) represent recent inbreeding events (Van Der Valk et al., 2019a). We used the physical length of ROHs as an approximation for genetic length and estimated that the long ROHs of 1-Mb trace back to <50 generations ago (*g* = 100 / 2 ^*^ ROH_Length_, where *g* is the number of generations and ROH_Length_ is the ROHs length in centimorgans) (Thompson, 2013). ROHs were identified for each individual using the “run of homozygosity” function in the program PLINK v1.90 (Purcell et al., 2007). We ran sliding windows of 20 SNPs on the VCF files of all genomes, requiring at least one SNP per 10 kb. In each individual genome, we allowed for the maximum of one heterozygous and 50 missing calls per windows.

The proportion of the genome within ROHs, i.e., genomic inbreeding coefficient (F_ROH_), was calculated as the total length of ROHs within an individual divided by the length of the genome (Mcquillan et al., 2008).

### Genetic Load Analyses

Deleterious mutations (genetic load) are predicted to disrupt gene function, and therefore expected to substantially reduce the mean fitness of individuals in a species/population (Mattila et al., 2012). To estimate genetic load in the endangered snub-nosed monkeys, we used SnpEff v.4.3t (Cingolani et al., 2012) to annotate variant sites (in our multisample VCF) based on the mappings and genome annotation of the golden snub-nosed monkey (Wang et al., 2019a) and to identify loss-of-function (LOF), missense, and synonymous mutations. The major homozygous alleles (allele frequency above 0.5) in each species and also in at least one of the outgroup species were used to represent the ancestral state. As an indicator of mutational load, for each individual, we counted the number of genes containing one or more homozygous-derived LOF and the total number of homozygous-derived missense mutations divided by the number of synonymous mutations (Fay et al., 2001; Van Der Valk et al., 2020), respectively.

We identified candidate genes with deleterious mutations for each species/population as those satisfying the following criteria: (*i*) mutations classified as LOF and (*ii*) mutations being homozygous and derived alleles.

## Results and Discussion

### Data, Sequencing, and SNP Calling

We analyzed a total of 106 individuals from all five snub-nosed monkey species and two outgroups, one *Pygathrix nemaeus* and one *Macaca mulatta* (Supplementary Table 1). Short reads from each individual were aligned to the high-quality chromosomal reference genome of the golden snub-nosed monkey (Wang et al., 2019a). An average mapping rate is 99.14% (78.73–100%), and average genomic depth is 12.02-fold coverage (5.04–35.25-fold coverage) (Supplementary Table 1). The high alignment rate and coverage ensures accurate identification of genetic variations. After stringent quality filtering, we identified a total of 108.28 million high-quality autosomal SNPs in the snub-nosed monkeys and the two outgroups.

Based on the kinship analyses, 44 potential consanguineous individuals (kinship coefficients > 0) were removed, resulting in a total of 62 individuals from all five snub-nosed monkeys used in the present analyses (kinship coefficients < 0). These included 40 individuals from *R. roxellana* (22 from SG, 13 from QL, and 5 from SNJ), 14 *R. bieti*, 4 *R. strykeri*, 2 *R. avunculus*, and 2 *R. brelichi* (Supplementary Table 1). After the removal of consanguineous individuals, we identified a total of 98.97 million high-quality autosomal SNPs in the 62 snub-nosed monkeys and the two outgroups.

### Genetic Diversity Analyses

**Figure 2:**
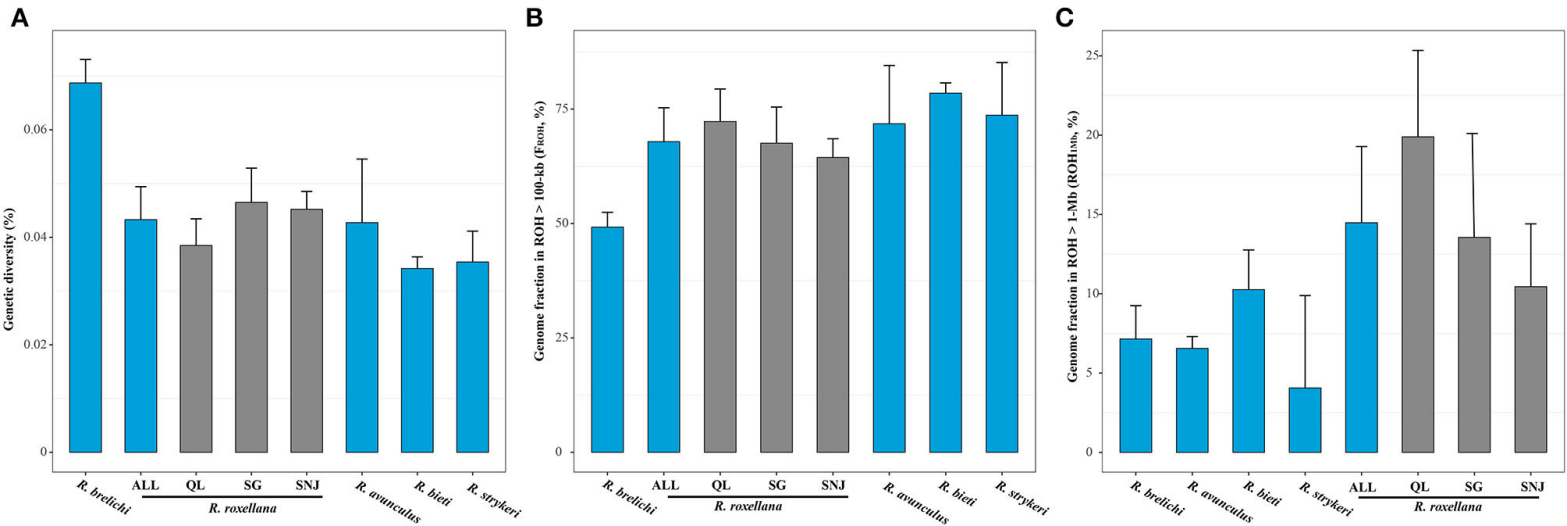
Genetic diversity and inbreeding level in the snub-nosed monkeys. Error bars represent standard deviation (sd). **(A)** The whole genome heterozygosity (*He*) of *R. brelich*i, *R. roxellana, R. avunculus, R. bieti*, and *R. strykeri*. Within *R. roxellana*: ALL, three populations; QL, Qinling population; SG, Sichuan/Gansu population; SNJ, Shennongjia population. **(B)** Fraction of the genomes in all ROHs (>100 kb) representing the genomic inbreeding coefficient (F_ROH_). **(C)** Fraction of genomes in long ROHs (>1 Mb; ROH_1Mb_) representing the recent inbreeding levels of the snub-nosed monkeys.

Whole genome heterozygosity (*He*) in snub-nosed monkeys ranged from 0.034 to 0.069% (Figure 2A). These estimates are similar to those obtained from other endangered and critically endangered primates, including aye-aye, Eastern lowland gorilla, mountain gorilla, Bornean orangutan, and pileated gibbon (0.051–0.073%) (Locke et al., 2011; Perry et al., 2012; Carbone et al., 2014; Xue et al., 2015), as well as estimated obtained from carnivores, including Bengal tiger, Amur tiger, white tiger, white lion, and African lion (0.040–0.073%) (Cho et al., 2013; Dobrynin et al., 2015). These estimates suggest that snub-nosed monkeys possess low genetic diversity, a pattern that may be related to recent environment change and human activities (Pan and Jablonski, 1987; Li et al., 2002; Zhou et al., 2016).

Contrary to the prevailing notion that smaller populations generally show lower levels of genetic diversity (Leffler et al., 2012; Cho et al., 2013; Prado Martinez et al., 2013; Díez-del-Molino et al., 2018), we found that *R. brelichi* possessed the highest genetic diversity (*He* = 0.069%; *p* < 0.05, Wilcox test; Figure 2A), despite its population size being lower (<400 individuals) than most other species. The unexpectedly high genetic diversity found in *R. brelichi* may be caused by interspecific hybridization (Zhou et al., 2014) or only small population remained from a large population in the recent past (Kolleck et al., 2013). *R. roxellana* (*He* = 0.043%) and *R. avunculus* (*He* = 0.042%) showed the second highest levels of genetic diversity, both higher than *R. bieti* (*He* = 0.034%) and *R. strykeri* (*He* = 0.036%) (*p* < 0.05, Wilcox test). Lower genetic diversity estimates for *R. bieti* and *R. strykeri* are consistent with their small population sizes (~3,000 individuals for *R. bieti* and 950 individuals for *R. strykeri*). The rather higher level of genetic diversity found in *R. roxellana* is consistent with their large population size (~22,500 individuals). We found, however, that although its small population size (<200 individuals; the lowest of all five snub-nosed monkey species), *R. avunculus* possesses relatively high genetic diversity, possibly due to ancient introgression or only small population remained from a large population in the recent past, similarly to *R. brelichi*. Intriguingly, the average pairwise nucleotide mismatches in 100-kb windows across the genome was 0.154% within *R. avunculus*, which is larger than in other snub-nosed monkeys (0.052–0.093%), suggesting a substantial amount of genetic divergence within the *R. avunculus* population. The two individuals of *R. avunculus* were potentially originated from two substantially diverged populations. These findings are consistent with field surveys that found at least two noncontiguous subpopulations in northern Vietnam (Boonratana and Le, 1998; Zhang et al., 2016). Our results indicate that these populations are highly divergent in *R. avunculus*, suggesting that they should be managed independently.

As for *R. brelichi* and *R. avunculus*, we found that the smallest population of *R. roxellana*, i.e., SNJ population (~1,200 individuals) also possessed relatively higher levels of genetic diversity (*He* = 0.044%) compared with the QL population (*He* = 0.038%, ~5,500 individuals, *p* = 0.0193, Wilcox test) and even similarly do for the largest population of *R. roxellana*, i.e., SG population (*He* = 0.046%, approximately 16,500 individuals, *p* = 0.5695, Wilcox test).

### Genomic Inbreeding and Recent Inbreeding Analyses

We found that genomic inbreeding coefficient (F_ROH_) of the snub-nosed monkeys to be between 49.37 and 78.86% (Figure 2B). Compared with other critically endangered or endangered species, the genomic inbreeding levels of the snub-nosed monkeys was higher than in the eastern gorillas (F_ROH_ = 34–39%) (Xue et al., 2015; Van Der Valk et al., 2019a), vervets (F_ROH_ = 6–12%) (Van Der Valk et al., 2019a), and pangolins (F_ROH_ = 12–42%) (Hu et al., 2020). These high levels of inbreeding of the snub-nosed monkeys are consistent with recent dramatic population decline (Zhou et al., 2016).

We found that *R. brelichi* (F_ROH_ = 49.37%) possessed the lowest level of genomic inbreeding and that *R. bieti* (F_ROH_ = 78.86%) and *R. strykeri* (F_ROH_ = 73.43%) have the highest level among the five snub-nosed monkey species (F_ROH_ = 68.11–78.86%) (*p* < 0.05, Wilcox test). Those of *R. roxellana* (F_ROH_ = 68.11%) and *R. avunculus* (F_ROH_ = 68.17%) are in between. As for heterozygosity levels, we found that the SNJ population of *R. roxellana* also possessed the lowest level of genomic inbreeding (F_ROH_ = 62.83%) compared with the SG (F_ROH_ = 67.24%, *p* = 0.2495, Wilcox test) and QL populations (F_ROH_ = 72.71%, *p* = 0.00262, Wilcox test). Analyses of long-ROHs (>1 Mb; ROH_1Mb_) indicated that although there are more individuals of *R. roxellana* than other snub-nosed monkey species, this species generally possessed more long-ROHs (ROH_1Mb_, 14.78%) than other species (*R. brelichi*, 7.41%; *R. bieti*, 10.85%; *R. strykeri*, 3.92%; and *R. avunculus*, 7.18%) (*p* < 0.05, Wilcox test), which all have smaller population sizes. Within *R. roxellana*, the SNJ with the smallest population size possessed the lowest recent inbreeding level (ROH_1Mb_, 10.19%) compared with the other two populations (SG, 13.01% and QL, 19.30%) (*p* < 0.05, Wilcox test) (Figure 2C).

It is generally thought that the smaller the population, the higher the inbreeding level (Keller and Waller, 2002). However, the results demonstrated that *R. brelichi, R. avunculus*, and the SNJ population of *R. roxellana*, which represented small populations, show high genetic diversity and low genomic and recent inbreeding. Thus, it seems that a potential mechanism could avoid inbreeding between close relatives, for example, the individual dispersal/transfer between social groups among these species/populations (Qi et al., 2009, 2014; Guo et al., 2010; Chang et al., 2014).

In comparison, *R. roxellana* has a large population size and demonstrates high genetic diversity and low genomic inbreeding, but high recent inbreeding. We speculate that the high recent inbreeding might result from the lower levels of population connectivity and habit utilization (~0.528–0.587 km^2^/individual) that was shaped by a recent increase in human activity for *R. roxellana* compared with the other snub-nosed monkeys (1–3.763 km^2^/individual; Supplementary Table 2) (Liu et al., 2015; Meyer et al., 2017; Guo et al., 2020). Thus, *R. roxellana* populations have experienced dozens of recent generations of close inbreeding in spite of their largest population size in the wild compared with other snub-nosed monkeys (Zhou et al., 2016).

### Genetic Load Analyses

**Figure 3:**
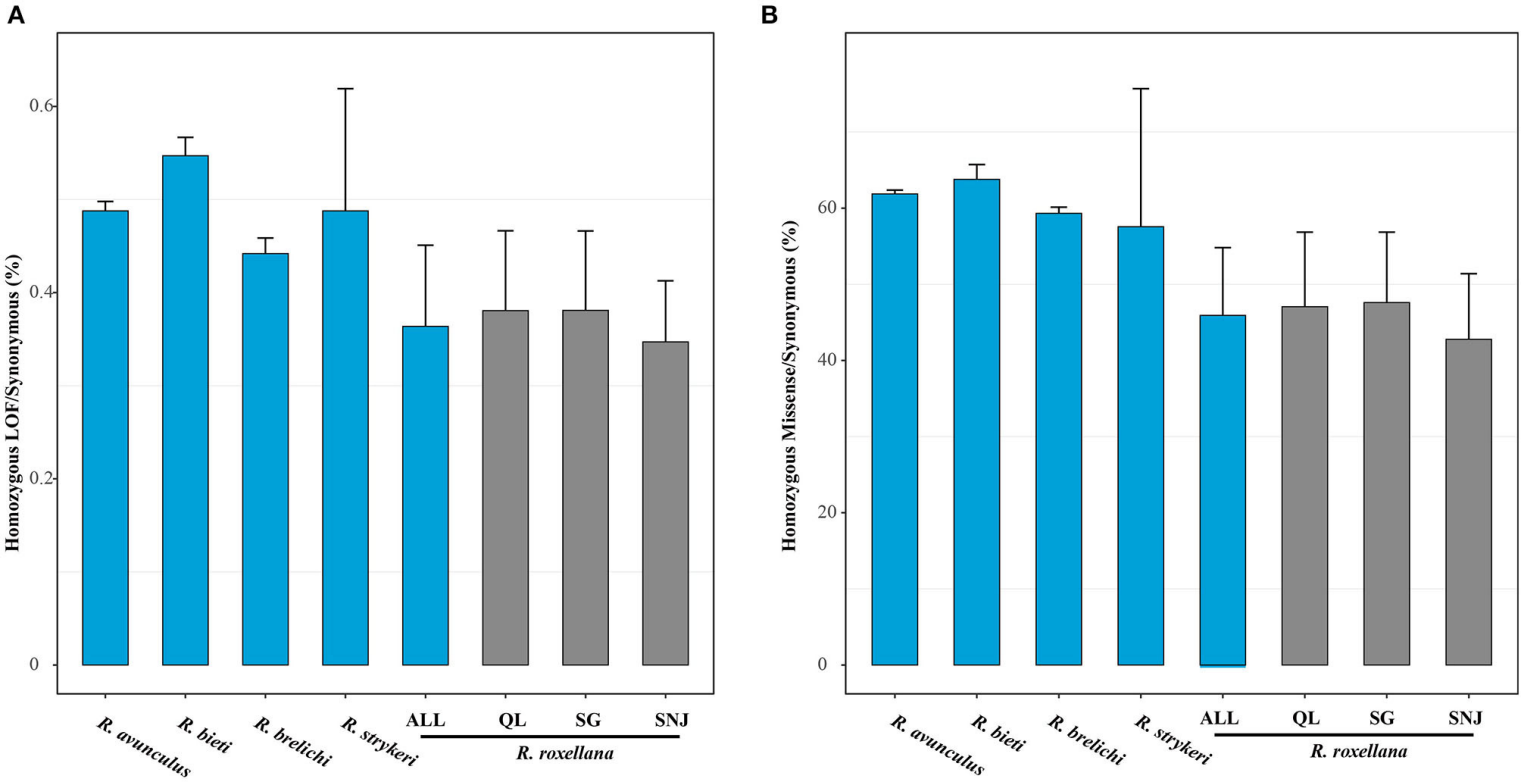
Genetic load of the snub-nosed monkeys. Error bars represent standard deviation (sd). **(A)** The ratios of the total numbers of homozygous-derived loss of function (LOF) to synonymous mutations of the species/populations. **(B)** The ratios of the homozygous-derived missense to synonymous mutations.

High levels of inbreeding can lead to increased homozygosity of recessive deleterious mutations, especially for small and isolated species/populations (Charlesworth and Willis, 2009), which will disrupt gene function or reduce individual fitness. We found that among the snub-nosed monkeys, individuals of *R. roxellana* with the highest recent inbreeding level carried significantly fewer homozygous-derived LOF (homozygous LOF/synonymous, 0.36%) than the other snub-nosed monkey species (*R. brelichi*: homozygous LOF/synonymous, 0.45%; *R. strykeri*: homozygous LOF/synonymous, 0.49%; *R. avunculus*: homozygous LOF/synonymous, 0.49%; and *R. bieti*: homozygous LOF/synonymous, 0.55%) (*p* < 0.001, Wilcox test) (Figure 3A). A similar pattern was observed when analyzing homozygous-derived missense mutations (Figure 3B). This suggests that purging homozygous-derived deleterious mutations is more efficient in *R. roxellana* compared with the other snub-nosed monkeys with smaller population sizes.

**Figure 4:**
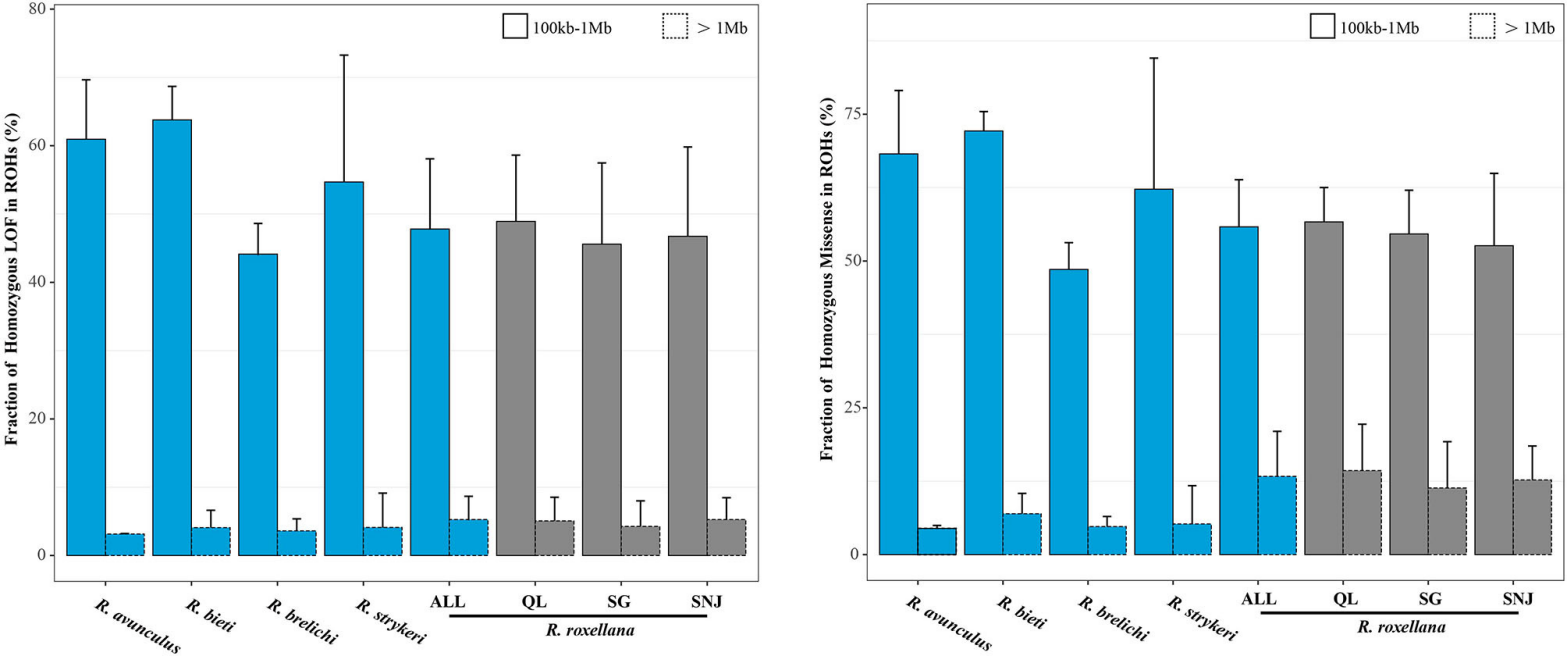
Fractions of homozygous LOF and missense mutations in the ROHs. Error bars represent standard deviation (sd). Bars with solid lines represent short ROHs (100 kb−1 Mb), and bars with dashed lines represent long ROHs (>1 Mb).

Our finding that *R. roxellana* possess high levels of inbreeding and relatively lower levels of mutational load results suggest that inbreeding do not necessarily lead to higher level of mutational load as previously suggested (Reed and Frankham, 2003), which is corroborated by the evidence that there is very low overlapping of homozygous-derived deleterious mutation regions with the long-ROH regions (4.86 and 12.90% overlapping between the homozygous-derived LOF and missense mutations with the long-ROHs, respectively), but rather more overlap with the short-ROHs regions (47.18 and 54.99%, respectively) (Figure 4). The present observation in *R. roxellana* that recent inbreeding did not lead to an excessive accumulation of homozygous-derived deleterious mutations was also recently reported in the snow leopard, island fox, and cheetah (Van Der Valk et al., 2019b). Interestingly, although the three populations of *R. roxellana* possessed highly different ROHs and heterozygosity profiles, we found that their levels of mutational load were remarkably similar (SNJ: homozygous LOF/synonymous, 0.33%; SG: homozygous LOF/synonymous, 0.38%; QL: homozygous LOF/synonymous, 0.38%) (*p* > 0.05, Wilcox test). Altogether, these findings support the idea that mutational load may build up over much longer time frame than inbreeding (Van Der Valk et al., 2019b).

Our findings of a significantly fewer homozygous-derived deleterious mutations in *R. roxellana* than in the other snub-nosed monkey species contradict that of a previous study, which identified similar numbers of LOF and derived missense mutations in *R. roxellana, R. bieti*, and *R. brelichi* (Zhou et al., 2016). These differences are likely the results of difference in sample sizes (*R. roxellana*: 40 in the present study vs. 26 in Zhou et al., 2016; *R. bieti*: 14 in the present study vs. 8 in Zhou et al., 2016; *R. brelichi*: 2 in the present study vs. 3 in Zhou et al., 2016) and the use of a high-quality chromosome-level reference genome assembly in the present study.

Many of the genes containing homozygous LOF mutations in the snub-nosed monkey genomes were associated with functions related to immunity (three genes in *R. roxellana*, nine genes in *R. bieti*, eight genes in *R. strykeri*, nine genes in *R. brelichi*, and nine genes in *R. avunculus*, Supplementary Table 3). For example, we found LOF mutations at the coagulation factor II receptor-like 3 gene (*F2RL3*), which is involved in the recruitment and behavior of immune cells and blood coagulation (Vergnolle et al., 2002; Leger et al., 2006; Gomides et al., 2012; Hossain et al., 2015), segregating in all five species. In addition, more immune genes with LOF mutations were found in the snub-nosed monkey species with the smaller population sizes (i.e., *R. bieti, R. strykeri, R. brelichi*, and *R. avunculus*). These include genes such as calmodulin-like protein 6 (*CALML6*) and lymphocyte-specific protein tyrosine kinase genes (*LCK*), both of which play a key role in the activation of T/B lymphocytes and the maintenance of balance of the immune system (Tewari et al., 2006; Wang et al., 2019b; Sheng et al., 2020). Altogether, this suggests that inbreeding depression may manifest in the form of lowered immune capability in the snub-nosed monkeys.

## Conclusion

Overall, this study provides new insights into the impact of population decline on genomic diversity in a set of highly endangered species, the snub-nosed monkeys (genus: *Rhinopithecus*). Our analyses demonstrated multiple counterintuitive patterns. For example, *R. brelichi, R. avunculus*, and SNJ population of *R. roxellana* with the small population size showed higher levels of genetic diversity, lower levels of genomic diversity, and recent inbreeding than other snub-nosed monkeys and other populations in *R. roxellana* with the larger population sizes. These findings suggest that, although their census population size is low, they have not yet lost much (if any) of their genetic variability over recent years. However, *R. roxellana* with the largest population size possesses high levels of recent breeding, despite low levels of genomic inbreeding and genetic load as well as high overall genetic diversity. This suggest that, despite its large population size, this species has likely been experiencing recent inbreeding, which has not yet affected its overall mutational load, and perhaps has not yet affected its fitness. Analyses of homozygous-derived deleterious mutations identified in all snub-nosed monkeys, however, suggest that these types of mutations are affecting immune, especially in smaller population sizes. This suggests that the long-term consequences of inbreeding may be resulting in an overall reduction of immune capability in the snub-nosed monkeys, which could provide a dramatic effect on their long-term survival prospects.

## Data Availability Statement

The datasets presented in this study can be found in online repositories. The names of the repository/repositories and accession number(s) can be found below: https://www.ncbi.nlm.nih.gov/ PRJNA616055.

## Ethics Statement

The animal study was reviewed and approved by the Committee on Animal Research and Ethics of Yunnan University (No. yuncare20200370).

## Author Contributions

LY designed the study. WK performed the data analyses. WK, LY, and QF wrote the manuscript. XF and QD performed the genomic DNA extraction and preparation of sequencing libraries. WX, CR, TN, LZ, and XY provided the samples. JH, HW, LF, DI, CR, and TN reviewed and revised the manuscript. All authors read and approved the final manuscript.

## Conflict of Interest

The authors declare that the research was conducted in the absence of any commercial or financial relationships that could be construed as a potential conflict of interest.
