# Supplementary material for: Genetic Diversity, Inbreeding Level, and Genetic Load in Endangered Snub-Nosed Monkeys (Rhinopithecus)
Source: Front Genet. 2020 Dec 15;11:615926. doi: 10.3389/fgene.2020.615926 (PMC7770136; doi:10.3389/fgene.2020.615926)
Supplement: Supplementary file 1 [file Data_Sheet_1.docx]

**Supplementary Information**

**Genetic Diversity, Inbreeding Level and Genetic Load in Endangered Snub-nosed Monkeys (*Rhinopithecus*)**

Weimin Kuang^1^, Jingyang Hu^1^, Hong Wu^1^, Xiaotian Fen^2,3,4^, Qingyan Dai^2,3,4^, Qiaomei Fu^2,3,4^, Wen Xiao^5^, Laurent Frantz^6,7^, Christian Roos^8^, Tilo Nadler^9^, David M Irwin^10^，Linchun Zhou^11^, Xu Yang^12^ and Li Yu^1*^

^1^State Key Laboratory for Conservation and Utilization of Bio-Resource in Yunnan, School of Life Sciences, Yunnan University, Kunming 650091, China,

^2^Key Laboratory of Vertebrate Evolution and Human Origins of Chinese Academy of Sciences, IVPP, CAS, Beijing 100044, China,

^3^Center for Excellence in Life and Paleoenvironment, Chinese Academy of Sciences, Beijing 100044, China,

^4^University of Chinese Academy of Sciences, Beijing 100049, China,

^5^Institute of Eastern-Himalaya Biodiversity Research, Dali University, Dali, Yunnan 671003, China,

^6^School of Biological and Chemical Sciences, Queen Mary University of London, London E1 4NS, UK,

^7^The Palaeogenomics and Bio-Archaeology Research Network, Department of Archaeology, University of Oxford, Oxford OX1 3TG, UK,

^8^Gene Bank of Primates and Primate Genetics Laboratory, German Primate Center, Leibniz Institute for Primate Research, Kellnerweg 4, 37077 Göttingen, Germany,

^9^Wildlife Consultant, Cuc Phuong Commune, Nho Quan District, Ninh Binh Province, Vietnam,

^10^Department of Laboratory Medicine and Pathobiology, University of Toronto, Toronto, Canada,

^11^Lushui Management and Conservation Branch of Gaoligong Mountain National Nature Reserve, Nujiang, China,

^12^Lushui Forestry and Grassland Council, Nujiang, 673100, China

***Correspondence to Li Yu:** yuli@ynu.edu.cn

**Supplementary Information includes:**

**Supplementary Notes**

**Supplementary Tables S1-S3**

**Supplementary Notes**

**DNA Extraction and Library preparation**

Bone and skin samples of *R. strykeri* and *R. avunculus* were used to extract DNA. For each sample, we shaved with a sterilized razor blade to remove the fur (for skin samples) and cut into small pieces of size <1 cubic millimeter, placing the pieces into a clean 2.0 mL PCR DNA LoBind tube (Eppendorf, cat. No. 30108078). Pieces of tissue for each sample were rinsed in 70% ethanol (Sigma Aldrich, cat. No. E7023) and then were vortexed at maximum speed for one minute, and then spun at 13,200 rpm in a table centrifuge for one minute. Finally, the resulting supernatant was removed. The mixing and spinning steps were repeated three times. Each tube was kept for five minutes at 40°C for complete ethanol evaporation. The remaining sample in each tube were used to prepare 50 uL of DNA extract per sample, using a modified DNA extraction method similar to that described in Dabney et al (2013).

Sequencing libraries were all treated with uracil-DNA-glycosylase (UDG) and endonuclease (Endo VIII) to remove characteristic ancient DNA deamination (Briggs et al., 2007). All of the extractions and library preparation were performed in a clean room at the Laboratory on Molecular Paleontology, at the Institute of Vertebrate Paleontology and Paleoanthropology (IVPP), Chinese Academy of Sciences, Beijing, China.

All sequencing libraries were PCR amplified using AccuPrimePfx DNA polymerase (Life Technologies) (Dabney and Meyer, 2012). Sample-specific indexes were introduced into both the P5 and P7 adaptors during this library amplification to allow differentiation of the samples after sequencing (Kircher et al., 2012). A NanoDrop 2000 spectrophotometer and a DNA-1000 chip on the Agilent Bioanalyzer 2100 were used to determine the library concentrations.

**Table S1**. Sample information and sequencing data summary of the snub-nosed monkeys and outgroups (rhesus macaque and red shanked douc langur) used in this study.

| **Sample_IDs** | **Species** | **Populations** | **Reference** | **Mapping Ratio** | **Depth** |
| --- | --- | --- | --- | --- | --- |
| Douc langur | *Pygathrix nemaeus* | Outgroup | This study | 99.50 | 32.13 |
| Rhesus macaque | *Macaca mulatta* | Outgroup | Zhang et al., 2014 | 98.03 | 27.90 |
| R.avunculus-1 | *R. avunculus* | NA | Zhou et al., 2014 | 98.32 | 26.39 |
| R.avunculus-2 | *R. avunculus* | NA | This study | 100.00 | 5.04 |
| R.bieti-1^a^ | *R. bieti* | NA | Zhou et al., 2016 | 99.72 | 8.72 |
| R.bieti-2 | *R. bieti* | NA | Zhou et al., 2016 | 99.70 | 9.10 |
| R.bieti-3 ^a^ | *R. bieti* | NA | Zhou et al., 2016 | 99.41 | 8.39 |
| R.bieti-4 ^a^ | *R. bieti* | NA | Zhou et al., 2016 | 99.61 | 8.51 |
| R.bieti-5 | *R. bieti* | NA | Zhou et al., 2016 | 99.56 | 9.54 |
| R.bieti-6 ^a^ | *R. bieti* | NA | Zhou et al., 2016 | 99.67 | 8.35 |
| R.bieti-7 | *R. bieti* | NA | Zhou et al., 2016 | 99.61 | 7.91 |
| R.bieti-8 ^a^ | *R. bieti* | NA | Zhou et al., 2016 | 99.70 | 10.64 |
| R.bieti-9 | *R. bieti* | NA | This study | 99.44 | 33.53 |
| R.bieti-10 | *R. bieti* | NA | Yu et al., 2016 | 99.38 | 7.99 |
| R.bieti-11 ^a^ | *R. bieti* | NA | Yu et al., 2016 | 97.62 | 10.94 |
| R.bieti-12 ^a^ | *R. bieti* | NA | Yu et al., 2016 | 99.38 | 9.04 |
| R.bieti-13 | *R. bieti* | NA | Yu et al., 2016 | 99.34 | 9.38 |
| R.bieti-14 | *R. bieti* | NA | Yu et al., 2016 | 99.52 | 9.15 |
| R.bieti-15 ^a^ | *R. bieti* | NA | Yu et al., 2016 | 99.37 | 9.49 |
| R.bieti-16 | *R. bieti* | NA | Yu et al., 2016 | 99.14 | 8.29 |
| R.bieti-17 ^a^ | *R. bieti* | NA | Yu et al., 2016 | 99.34 | 8.64 |
| R.bieti-18 | *R. bieti* | NA | Yu et al., 2016 | 99.31 | 9.58 |
| R.bieti-19 ^a^ | *R. bieti* | NA | Yu et al., 2016 | 99.27 | 8.72 |
| R.bieti-20 ^a^ | *R. bieti* | NA | This study | 99.63 | 10.11 |
| R.bieti-21 | *R. bieti* | NA | This study | 98.96 | 12.07 |
| R.bieti-22 ^a^ | *R. bieti* | NA | Yu et al., 2016 | 99.70 | 10.84 |
| R.bieti-23 | *R. bieti* | NA | This study | 98.22 | 11.49 |
| R.bieti-24 ^a^ | *R. bieti* | NA | Yu et al., 2016 | 99.22 | 8.72 |
| R.bieti-25 ^a^ | *R. bieti* | NA | This study | 99.71 | 11.56 |
| R.bieti-26 | *R. bieti* | NA | Yu et al., 2016 | 99.22 | 9.09 |
| R.bieti-27 ^a^ | *R. bieti* | NA | Yu et al., 2016 | 99.34 | 8.60 |
| R.bieti-28 | *R. bieti* | NA | This study | 99.72 | 8.85 |
| R.bieti-29 | *R. bieti* | NA | Yu et al., 2016 | 98.74 | 8.99 |
| R.bieti-30 | *R. bieti* | NA | Yu et al., 2016 | 99.04 | 9.47 |
| R.bieti-31 | *R. bieti* | NA | Yu et al., 2016 | 99.45 | 10.99 |
| R.bieti-32 ^a^ | *R. bieti* | NA | This study | 99.72 | 11.11 |
| R.bieti-33 ^a^ | *R. bieti* | NA | Yu et al., 2016 | 99.12 | 8.72 |
| R.bieti-34 ^a^ | *R. bieti* | NA | Yu et al., 2016 | 99.31 | 10.44 |
| R.bieti-35 | *R. bieti* | NA | Yu et al., 2016 | 97.00 | 22.94 |
| R.brelichi-1 ^a^ | *R. brelichi* | NA | Zhou et al., 2016 | 99.46 | 7.64 |
| R.brelichi-2 | *R. brelichi* | NA | Zhou et al., 2016 | 99.59 | 9.11 |
| R.brelichi-3 ^a^ | *R. brelichi* | NA | Zhou et al., 2016 | 99.68 | 11.73 |
| R.brelichi-4 | *R. brelichi* | NA | Yu et al., 2016 | 99.26 | 30.58 |
| R.brelichi-5 ^a^ | *R. brelichi* | NA | Zhou et al., 2014 | 99.65 | 21.86 |
| R.roxellana-1 ^a^ | *R. roxellana* | SG | Zhou et al., 2016 | 99.77 | 10.29 |
| R.roxellana-2 | *R. roxellana* | SG | Zhou et al., 2016 | 99.76 | 7.87 |
| R.roxellana-3 ^a^ | *R. roxellana* | SG | Zhou et al., 2016 | 99.74 | 24.53 |
| R.roxellana-4 | *R. roxellana* | SG | Zhou et al., 2016 | 99.72 | 7.89 |
| R.roxellana-5 | *R. roxellana* | SG | Zhou et al., 2016 | 99.74 | 8.85 |
| R.roxellana-6 | *R. roxellana* | SG | Zhou et al., 2016 | 99.66 | 8.23 |
| R.roxellana-7 | *R. roxellana* | SG | Zhou et al., 2016 | 99.72 | 10.90 |
| R.roxellana-8 | *R. roxellana* | SG | Zhou et al., 2016 | 99.74 | 9.66 |
| R.roxellana-9 | *R. roxellana* | SG | Zhou et al., 2016 | 99.80 | 9.98 |
| R.roxellana-10 | *R. roxellana* | SG | Zhou et al., 2016 | 99.63 | 7.94 |
| R.roxellana-11 | *R. roxellana* | SG | Zhou et al., 2016 | 99.64 | 8.10 |
| R.roxellana-12 | *R. roxellana* | SG | Zhou et al., 2016 | 99.62 | 9.33 |
| R.roxellana-13 | *R. roxellana* | SG | Zhou et al., 2016 | 99.69 | 8.67 |
| R.roxellana-14 | *R. roxellana* | QL | Zhou et al., 2016 | 99.04 | 7.50 |
| R.roxellana-15 | *R. roxellana* | QL | Zhou et al., 2016 | 99.74 | 7.55 |
| R.roxellana-16 | *R. roxellana* | QL | Zhou et al., 2016 | 99.73 | 7.98 |
| R.roxellana-17 | *R. roxellana* | QL | Zhou et al., 2016 | 99.77 | 8.13 |
| R.roxellana-18 | *R. roxellana* | QL | Zhou et al., 2016 | 99.76 | 10.43 |
| R.roxellana-19 | *R. roxellana* | QL | Zhou et al., 2016 | 98.42 | 24.30 |
| R.roxellana-20 | *R. roxellana* | QL | Zhou et al., 2016 | 99.64 | 10.59 |
| R.roxellana-21 | *R. roxellana* | SNJ | Zhou et al., 2016 | 99.77 | 24.09 |
| R.roxellana-22 | *R. roxellana* | SNJ | Zhou et al., 2016 | 99.59 | 9.08 |
| R.roxellana-23 ^a^ | *R. roxellana* | SNJ | Zhou et al., 2016 | 99.55 | 10.56 |
| R.roxellana-24 ^a^ | *R. roxellana* | SNJ | Zhou et al., 2016 | 99.73 | 12.24 |
| R.roxellana-25 | *R. roxellana* | SNJ | Zhou et al., 2016 | 99.76 | 10.64 |
| R.roxellana-26 ^a^ | *R. roxellana* | SNJ | Zhou et al., 2016 | 99.67 | 9.71 |
| R.roxellana-27 ^a^ | *R. roxellana* | SNJ | Yu et al., 2016 | 99.45 | 29.55 |
| R.roxellana-28 | *R. roxellana* | SG | Yu et al., 2016 | 99.18 | 9.90 |
| R.roxellana-29 ^a^ | *R. roxellana* | SNJ | Kuang et al., 2019 | 99.66 | 12.06 |
| R.roxellana-30 ^a^ | *R. roxellana* | SNJ | Yu et al., 2016 | 99.73 | 12.34 |
| R.roxellana-31 | *R. roxellana* | SNJ | Kuang et al., 2019 | 98.57 | 12.75 |
| R.roxellana-32 ^a^ | *R. roxellana* | SNJ | Yu et al., 2016 | 97.99 | 11.13 |
| R.roxellana-33 | *R. roxellana* | SNJ | Yu et al., 2016 | 99.50 | 8.76 |
| R.roxellana-34 ^a^ | *R. roxellana* | SNJ | Yu et al., 2016 | 99.57 | 8.94 |
| R.roxellana-35 | *R. roxellana* | SG | Yu et al., 2016 | 99.40 | 10.99 |
| R.roxellana-36 | *R. roxellana* | QL | Kuang et al., 2019 | 98.77 | 8.64 |
| R.roxellana-37 ^a^ | *R. roxellana* | QL | Yu et al., 2016 | 95.73 | 9.00 |
| R.roxellana-38 | *R. roxellana* | SG | Yu et al., 2016 | 99.76 | 10.50 |
| R.roxellana-39 | *R. roxellana* | QL | Yu et al., 2016 | 99.18 | 8.82 |
| R.roxellana-40 ^a^ | *R. roxellana* | QL | Yu et al., 2016 | 99.80 | 10.78 |
| R.roxellana-41 | *R. roxellana* | QL | Yu et al., 2016 | 99.77 | 10.14 |
| R.roxellana-42 | *R. roxellana* | QL | Yu et al., 2016 | 99.75 | 11.03 |
| R.roxellana-43 ^a^ | *R. roxellana* | QL | Yu et al., 2016 | 99.77 | 9.70 |
| R.roxellana-44 ^a^ | *R. roxellana* | QL | Yu et al., 2016 | 99.67 | 8.10 |
| R.roxellana-45 | *R. roxellana* | QL | Yu et al., 2016 | 99.68 | 8.70 |
| R.roxellana-46 | *R. roxellana* | QL | Yu et al., 2016 | 98.25 | 11.24 |
| R.roxellana-47 | *R. roxellana* | SG | Yu et al., 2016 | 99.61 | 11.44 |
| R.roxellana-48 | *R. roxellana* | SG | Yu et al., 2016 | 98.16 | 10.16 |
| R.roxellana-49 | *R. roxellana* | SG | Yu et al., 2016 | 99.69 | 10.78 |
| R.roxellana-50 ^a^ | *R. roxellana* | SG | Yu et al., 2016 | 99.64 | 10.68 |
| R.roxellana-51 | *R. roxellana* | SG | Yu et al., 2016 | 99.73 | 8.35 |
| R.roxellana-52 ^a^ | *R. roxellana* | SG | Yu et al., 2016 | 99.76 | 11.23 |
| R.roxellana-53 ^a^ | *R. roxellana* | SG | Yu et al., 2016 | 99.76 | 9.17 |
| R.roxellana-54 | *R. roxellana* | SG | Kuang et al., 2019 | 99.63 | 9.84 |
| R.roxellana-55 | *R. roxellana* | SG | Kuang et al., 2019 | 99.08 | 8.69 |
| R.roxellana-56 | *R. roxellana* | SG | Yu et al., 2016 | 99.79 | 9.61 |
| R.roxellana-57 | *R. roxellana* | SG | Zhou et al., 2014 | 99.75 | 30.48 |
| R.strykeri-1 ^a^ | *R. strykeri* | NA | Zhou et al., 2016 | 99.67 | 9.76 |
| R.strykeri-2 | *R. strykeri* | NA | Yu et al., 2016 | 99.52 | 35.25 |
| R.strykeri-3 | *R. strykeri* | NA | Yu et al., 2016 | 93.81 | 24.08 |
| R.strykeri-4 ^a^ | *R. strykeri* | NA | Zhou et al., 2014 | 78.73 | 18.98 |
| R.strykeri-5 ^a^ | *R. strykeri* | NA | This study | 100.00 | 9.89 |
| R.strykeri-6 | *R. strykeri* | NA | This study | 100.00 | 10.00 |
| R.strykeri-7 | *R. strykeri* | NA | This study | 100.00 | 9.18 |

Note: ^a^ Potential consanguineous samples identified by the kinship analyses (kinship coefficients > 0) using KING v.2.2.5 software

**Table S2.** Summary of size of the habitat range and numbers of wild individual of the five snub-nosed monkeys species. Data are from Liu et al. (2016), Meyer et al. (2017), Guo et al. (2020) and IUCN (2020).

| Species | Population | Individuals | Habitat range (km²) | km²/individual |
| --- | --- | --- | --- | --- |
| *R. roxellana* | SNJ | 1200 | 704.67 | 0.587 |
|  | QL | 5500 | 2905.17 | 0.528 |
|  | SG | 16500 | 8807.01 | 0.534 |
| *R. bieti* | | 3000 | 4813.75 | 1.605 |
| *R. brelichi* | | 400 | 514 | 1.285 |
| *R. strykeri* | | 950 | 3575 | 3.763 |
| *R. avunculus* | | 200 | 200 | 1.000 |

**Table S3.** Immunity related genes identified with homozygous deleterious-derived mutations for each species of the snub-nosed monkeys.

| Species | Immunity Related Genes identified |
| --- | --- |
| *R. roxellana* | *F2RL3 NLRP10 VAV3* |
| *R. brelichi* | *F2RL3 CALML6 LCK FCRL4 LRRFIP2 MSR1 NOD2 SIRPB1 ZBP1* |
| *R. bieti* | *F2RL3 ABCG1 BPIFB1 CALML6 LCK MSR1 NCF1 NR1H3 SIGLEC16* |
| *R. avunculus* | *F2RL3 ABCG1 BPIFB1 CALML6* *LCK MSR1 NLRC4 SMAP2 SIGLEC16* |
| *R. stryker* | *F2RL3 ABCG1 BPIFB1 CALML6 LCK MSR1 NR1H3 SIGLEC16* |

Note: The immunity related gene found in all species of snub-nosed monkeys is shaded. The immunity related genes found in snub-nosed monkey species with small population sizes (i.e., *R. bieti*, *R. strykeri*, *R. brelichi* and *R. avunculus*) are underlined.

**REFERENCES**

Briggs, A.W., Stenzel, U., Johnson, P.L., Green, R.E., Kelso, J., Prufer, K., et al. (2007). Patterns of damage in genomic DNA sequences from a Neandertal. *Proc Natl Acad Sci USA* 104**,** 14616-14621.doi: 10.1073/pnas.0704665104

Dabney, J., Knapp, M., Glocke, I., Gansauge, M.T., Weihmann, A., Nickel, B., et al. (2013). Complete mitochondrial genome sequence of a Middle Pleistocene cave bear reconstructed from ultrashort DNA fragments. *Proc Natl Acad Sci USA* 110**,** 15758-15763.doi: 10.1073/pnas.1314445110

Dabney, J., and Meyer, M. (2012). Length and GC-biases during sequencing library amplification: A comparison of various polymerase-buffer systems with ancient and modern DNA sequencing libraries. *Biotechniques* 52**,** 87-94.doi: 10.2144/000113809

Guo, Y., Ren, B., Dai, Q., Zhou, J., Paul, A.G., and Zhou, J. (2020). Habitat estimates reveal that there are fewer than 400 Guizhou snub-nosed monkeys, *Rhinopithecus brelichi*, remaining in the wild. *Glob Ecol and Conserv*, e01181.doi: 10.1016/j.gecco.2020.e01181

Kircher, M., Sawyer, S., and Meyer, M. (2012). Double indexing overcomes inaccuracies in multiplex sequencing on the Illumina platform. *Nucleic Acids Res* 40**,** e3.doi: 10.1093/nar/gkr771

Liu, Z., Liu, G., Roos, C., Wang, Z., Xiang, Z., Zhu, P., et al. (2015). Implications of genetics and current protected areas for conservation of 5 endangered primates in China. *Conserv Biol* 29**,** 1508-1517.doi: 10.1111/cobi.12581

Meyer, D., Momberg, F., Matauschek, C., Oswald, P., Lwin, N., Aung, S., et al. (2017). Conservation status of the Myanmar or Black snub-nosed monkey *Rhinopithecus strykeri*. Fauna & Flora International, Yangon, Myanmar; Institute of Eastern-Himalaya Biodiversity Research, Dali, China; and German Primate Center, Göttingen, Germany
